# Prediction of three lipid derivatives for postoperative gastric cancer mortality: the Fujian prospective investigation of cancer (FIESTA) study

**DOI:** 10.1186/s12885-018-4596-y

**Published:** 2018-08-06

**Authors:** Dan Hu, Feng Peng, Xiandong Lin, Gang Chen, Binying Liang, Ying Chen, Chao Li, Hejun Zhang, Guohui Fan, Guodong Xu, Yan Xia, Jinxiu Lin, Xiongwei Zheng, Wenquan Niu

**Affiliations:** 1 Department of Pathology, Fujian Cancer Hospital & Fujian Medical University Cancer Hospital, No.420 Fu Ma Road, Jin An District, Fuzhou, 350014 Fujian China; 2 Department of Cardiology, The First Affiliated Hospital of Fujian Medical University, Fuzhou, Fujian China; 3 Department of Medical Record, Fujian Cancer Hospital & Fujian Medical University Cancer Hospital, Fuzhou, Fujian China; 4 Department of Core Research Laboratory, Fujian Cancer Hospital & Fujian Medical University Cancer Hospital, Fuzhou, Fujian China; 5 Institute of Clinical Medical Sciences, China-Japan Friendship Hospital, No.2 Yinghua East Street, Chao Yang District, Beijing, 100029 China

**Keywords:** The FIESTA study, Gastric cancer, Lipid derivative, Metabolic syndrome, Mortality, Prognosis

## Abstract

**Background:**

As we previously reported, the presence of preoperative metabolic syndrome can predict the significant risk of gastric cancer mortality. As a further extension, we evaluated the prediction of three lipid derivatives generated from triglycerides (TG), total cholesterol (TC), high- and low-density lipoprotein cholesterol (HDLC and LDLC) at baseline for postoperative gastric cancer mortality by prospectively analysing 3012 patients. The three lipid derivatives included the ratio of TC minus HDLC to HDLC known as atherogenic index (AI), the ratio of TG to HDLC abbreviated as THR and the ratio of LDLC to HDLC abbreviated as LHR.

**Methods:**

Gastric cancer patients who received gastrectomy between January 2000 and December 2010 were consecutively recruited from Fujian Cancer Hospital. Follow-up assessment was implemented annually before December 2015.

**Results:**

Finally, there were 1331 deaths from gastric cancer and 1681 survivors, with a median follow-up time of 44.05 months. 3012 patients were evenly randomized into the derivation group and the validation group, and both groups were well balanced at baseline. Overall adjusted estimates in the derivation group were statistically significant for three lipid derivatives (hazard ratio [HR]: 1.20, 1.17 and 1.19 for AI, THR and LHR, respectively, all *P* < 0.001), and were reproducible in the validation group. The risk prediction of three lipid derivatives was more obvious in males than females, in patients with tumor-node-metastasis stage I-II than stage III-IV, in patients with intestinal-type than diffuse-type gastric cancer, in patients with normal weight than obesity, and in patients without hypertension than with hypertension, especially for AI and LHR, and all results were reproducible. Calibration and discrimination statistics showed good reclassification performance and predictive accuracy when separately adding three lipid derivatives to baseline risk model. A prognostic nomogram was accordingly built based on significant attributes to facilitate risk assessment, with a good prediction capability.

**Conclusions:**

Our results indicate that preoperative lipid derivatives, especially AI and LHR, are powerful predictors of postoperative gastric cancer mortality, with more obvious prediction in patients of male gender or with tumor-node-metastasis stage I-II or intestinal-type gastric cancer, and in the absence of obesity or hypertension before gastrectomy.

**Electronic supplementary material:**

The online version of this article (10.1186/s12885-018-4596-y) contains supplementary material, which is available to authorized users.

## Background

Gastric cancer is one of the most common malignancies worldwide [1]. In China, national statistics show that gastric cancer is the second-leading cancer killer, and it took the lives of around 498,000 people in 2015 [2]. At present, early detection of gastric cancer currently poses a challenge, because most patients are asymptomatic at early stages, but progress to advanced stages once diagnosed [3, 4]. Thus far, surgery is a preferred choice to treat patients with resectable gastric cancer [5]. However, for some patients, the prognosis after gastrectomy or adjuvant treatment is unsatisfactory, with an overall 5-year survival rate of less than 25% [6]. So, how to better predict the prognosis of postoperative gastric cancer patients thus far becomes a problem demanding prompt solutions [7, 8]. More recently, we, in an ongoing Fujian prospective investigation of cancer (FIESTA) study, analysed 3012 gastric cancer patients who were postoperatively monitored from 1.1 months to 183.3 months, and found that preoperative metabolic syndrome can significantly predict gastric cancer mortality after gastrectomy, to which systolic blood pressure, fasting blood glucose, triglyceride (TG) and high-density lipoprotein cholesterol (HDLC) contributed remarkably [9]. In this present study, to provide an in-depth evaluation of dyslipidaemia - an integral element of the metabolic syndrome, we generated three lipid derivatives from TG, total cholesterol (TC), HDLC and low-density lipoprotein cholesterol (LDLC) as prognostic predictors for gastric cancer mortality among 3012 patients after gastrectomy in the FIESTA database. The three lipid derivatives included the ratio of TC minus HDLC to HDLC known as atherogenic index (AI), the ratio of TG to HDLC abbreviated as THR and the ratio of LDLC to HDLC abbreviated as LHR, and they have been widely evaluated in association with cardiovascular diseases and the prediction capability is superior to individual lipids [10, 11]. It remains uncertain, however, whether these lipid derivatives at baseline can effectively predict the prognosis of postoperative gastric cancer.

## Methods

### The FIESTA study

The FIESTA study is an ongoing exploration of preoperative factors for predicting disease-specific mortality of common digestive tract cancer, including sites at esophagus [12–15], stomach [9, 16] and colon and rectum [17–20]. The study proposal was approved by the Ethics Committee of Fujian Cancer Hospital. All patients gave written informed consent.

### Study patients

Patients with gastric cancer were consecutively recruited from the Department of Thoracic Surgery, Fujian Cancer Hospital between January 2000 and December 2010. Only patients who received total gastrectomy, distal partial gastrectomy or proximal partial gastrectomy based on the position and size of tumors were includable. Moreover, they reported no consanguinity and were of Han Chinese descent. Furthermore, they received gastrectomy for the first time, and had not received preoperative and postoperative chemotherapy or radiotherapy. Initially, a total of 3413 qualified patients were followed up.

### Follow-up assessment

Postoperative patients who were discharged from Fujian Cancer Hospital were followed up annually prior to December 2015, unless lost to follow-up, dropped out of study or dead before that time. Follow-up assessment was conducted at the Out-Patient Department by contacting patients through phone calls or postal letters if they had missed appointments. The minimal follow-up was 1 month to avoid deaths from unclear surgical complications. Case-specific mortality from gastric cancer was the primary outcome. Survival time in months was calculated from the date of initial admission to the date of death or last follow-up visit, whichever occurred fist. Finally, 118 patients were lost to follow-up, 48 patients were monitored less than 1 month and 235 patients died of causes from non-gastric cancer, leaving 3012 patients in the present analysis. As of December 31, 2015, overall median follow-up time was 44.05 months (range: 1.1 months to 183.3 months), and 1331 deaths from gastric cancer occurred, leaving 1681 survivors. The minimal follow-up time of all patients was 5 years, which ensured sufficient power to predict postoperative survival at 5-year time point.

### Specimens

Fasting venous blood specimens were drawn the day of receiving gastrectomy. Plasma TG, TC, and HDLC and LDLC were measured per standard procedures at the Clinical Laboratory of Fujian Cancer Hospital. Fasting blood glucose (FBG) was determined by an automated glucose-oxidase method.

Primary gastric cancer tissue and adjacent normal tissue specimens were surgically resected from all patients during the period of gastrectomy. All tissue samples were formalin-fixed, paraffin-embedded and frozen within 1 h after tumor removal.

### Baseline characteristics

After signing informed consent, each patient was invited to complete a structured questionnaire covering information on date of birth, onset age of gastric cancer, gender, smoking status, drinking status and family history of cancer. Weight and height were measured at the time of admission when patients wore light clothing and no shoes. Body mass index (BMI) was calculated as the weight (kg)/height (m)^2^. Blood pressure (BP) was measured on three occasions at roughly 5-min intervals using a mercury sphygmomanometer by certified nurses, while patients were in seated position per the standard protocol recommended by the American Heart Association [21]. Age of patients at the time of receiving gastrectomy was recorded. Smoking status was categorized into never smoking and ever (former/current) smoking. Drinking status was categorized into never drinking and ever (former/current) drinking. Family cancer history was reported if one or more of affected relatives within three generations who suffered from malignancies except non-melanoma skin cancer.

Obesity was defined as BMI ≥25 kg/m^2^. Hypertension was defined as systolic BP ≥140 mmHg or diastolic BP ≥90 mmHg or use of antihypertensive medications. Diabetes was defined as FBG ≥7.0 mmol/L.

### Clinicopathologic characteristics

Clinicopathologic characteristics were elicited from medical charts and pathological reports of patients, including tumor-node-metastasis (TNM) stage (I-IV) [22], tumor size (in centimeters), depth of invasion (T1-T4), regional lymph node metastasis (N0-N3), distant metastasis (M0 and M1), Lauren’s classification (intestinal type and diffuse type), number of lymph node metastasis (LNM) and tumor embolus.

### Statistics

To derive a reproducible estimate, all 3012 patients were evenly randomized into the derivation group and the validation group. Gastric cancer mortality was calculated in both groups. Continuous variables were presented as mean (standard deviation or SD) or median (interquartile range), and their comparisons between the derivation and validation groups were implemented with the t-test or the Mann-Whitney test if appropriate. Categorical variables were presented as percentage (count) and compared with the [Chi]^2^ test. Weibull proportional hazards regression analysis was performed to estimate hazard ratio with its 95% confidence interval (95% CI) of each lipid derivative for gastric cancer mortality with and without considering confounders. For binary lipid derivatives, Kaplan-Meier curves were used to display changes of cumulative survival rates against follow-up time, and Log-rank tests were used to compare differences in median survival time (MST).

Predictive accuracy was evaluated with discrimination and calibration statistics. Discrimination statistics included Harrell C-statistic to see whether the addition of individual lipid derivatives can differentiate among patients who died from gastric cancer or survived. Calibration statistics included Akaike information criterion (AIC) and Bayesian information criterion (BIC), and the − 2 log likelihood ratio tests were used to assess how closely prediction probability by adding lipid derivatives reflected the actual observed risk and global fit of modified risk model.

In addition, net benefit gained by adding individual lipid derivatives to traditional risk model was displayed as a curve in decision curve analysis [23]. The X-axis of this curve represents thresholds for gastric cancer mortality, and the Y-axis represents net benefits hinged on different thresholds. A higher net benefit is shown if the “model” curve is farther away from solid curve line when assuming all gastric cancer mortality and dotted horizontal line when assuming none gastric cancer mortality. The farthest the curve is, the highest the net benefit is.

Finally, a prognostic nomogram displaying 3-year, 5-year and 10-year gastric cancer mortality was constructed for clinical application among all study patients. The attributes in nomogram were selected from baseline demographic, clinical and clinicopathologic variables that were significantly associated with gastric cancer mortality besides three lipid derivatives. The predictive accuracy and discriminative capability of this nomogram were assessed by the concordance index (C-index) and calibration curves. The C-index ranges from 0.0 to 1.0, with the higher value indicating a higher accuracy. It is generally accepted that the C-index of less than 0.7 suggests no improvement in model performance [24]. In calibration curve, the 45-degree line represents optimal predictions, and it illustrates how far the predicted probabilities of this nomogram are from actual observations. The prognostic nomogram was generated by the regression modeling strategies (RMS) package (https://cran.r-project.org/web/packages/rms/index.html) in the R-language (version 3.3.3).

Unless otherwise stated, the STATA/SE software (version 14.0, StataCorp, TX, USA) was employed for data management and statistical analyses. *P* value less than 0.05 was considered statistically significant.

## Results

### Baseline characteristics

Baseline demographic, clinical and clinicopathologic characteristics were compared between the derivation and validation groups, as shown in Table 1. Both groups had an equal number of study patients. All characteristics were comparable between the two groups, except gender composition (*P* = 0.051).

**Table 1:** Comparisons of baseline demographic, clinical and clinicopathologic characteristics between the derivation and validation groups

Characteristics	Derivation group	Validation group	*P*
Number	1506	1506	
Age (years)	58.47 (11.09)	58.76 (11.30)	0.503
Males	72.78% (1096)	75.9% (1143)	0.051
Ever smokers	18.39% (277)	18.53% (279)	0.925
Ever drinkers	5.84% (88)	5.31% (80)	0.525
Family cancer history (+)	8.43% (127)	8.7% (131)	0.795
Body mass index (kg/m^2^)	22.72 (3.14)	22.91 (3.01)	0.104
Systolic blood pressure (mmHg)	124.16 (19.03)	124.84 (19.92)	0.353
Diastolic blood pressure (mmHg)	76.85 (11.37)	77.43 (11.51)	0.179
Fasting blood glucose (mmol/L)	6.21 (2.48)	6.19 (2.43)	0.853
AI	3.95 (2.28)	3.88 (1.93)	0.209
THR	1.41 (1.47)	1.36 (1.27)	0.339
LHR	3.30 (1.79)	3.28 (1.56)	0.505
TNM stage			0.122
I	12.44% (177)	11.76% (167)	
II	15.95% (227)	13.87% (197)	
III	54.67% (778)	59.01% (838)	
IV	16.94% (241)	15.35% (218)	
Lauren’s classification			0.551
Intestinal type	39.77% (552)	38.04% (528)	
Diffuse type	60.23% (836)	61.96% (860)	
Tumor embolus			0.976
Positive	39.01% (541)	38.95% (541)	
Negative	60.99% (846)	61.05% (848)	
Tumor size (cm)	5.55 (2.85); 5 (3.5, 7)	5.66 (3.13); 5 (3.5, 7)	0.337
Number of lymph node metastasis	5.60 (6.91); 3 (0, 8)	5.70 (7.10); 3 (0, 8)	0.724

Data are represented as mean (standard deviation) or median (interquartile range) or percentage (count)

*Abbreviations*: *AI* atherogenic index, *THR* the triglyceride to high-density lipoprotein cholesterol ratio, *LHR* the low-density lipoprotein cholesterol to high-density lipoprotein cholesterol ratio, *TNM* tumor-node-metastasis

### Risk estimates

The risk estimates of three lipid derivatives were calculated based on per SD increment of AI (SD: 2.12), THR (SD: 1.38) and LHR (SD: 1.68) among all study patients. Overall and stratified risk estimates of three lipid derivatives in both groups for gastric cancer mortality are summarized in Table 2. Relative to unadjusted overall estimates, consideration of possible confounding factors attenuated risk estimates for all three lipid derivatives in both groups, yet still being remarkably significant.

**Table 2:** Overall and stratified risk estimates of three lipid derivatives for gastric cancer mortality in both derivation and validation groups

Characteristics	Subgroups	Derivation group (*n* = 1506)			Validation group (*n* = 1506)		
		AI	THR	LHR	AI	THR	LHR
Overall	Unadjusted	1.28, 1.22–1.35, < 0.001	1.19, 1,14–1.24, < 0.001	1.24, 1.19–1.30, < 0.001	1.32, 1.23–1.41, < 0.001	1.21, 1.13–1.29, < 0.001	1.29, 1.21–1.38, < 0.001
Overall	Adjusted	1.20, 1.13–1.26, < 0.001	1.17, 1.11–1.24, < 0.001	1.19, 1.13–1.25, < 0.001	1.27, 1.17–1.37, < 0.001	1.16, 1.08–1.24, < 0.001	1.24, 1.15–1.35, < 0.001
Gender	Male	1.27, 1.17–1.38, < 0.001	1.18, 1.11–1.26, < 0.001	1.25, 1.15–1.36, < 0.001	1.30, 1.18–1.43, < 0.001	1.17, 1.08–1.27, < 0.001	1.26, 1.16–1.38, < 0.001
	Female	1.16, 1.06–1.26, 0.001	1.17, 1.03–1.33, 0.019	1.19, 1.10–1.29, < 0.001	1.26, 1.06–1.48, 0.012	1.12, 0.97–1.29, 0.136	1.17, 0.96–1.42, 0.118
Smoking	Ever	1.15, 0.90–1.47, 0.133	1.14, 1.05–1.24, 0.011	1.14, 0.90–1.45, 0.270	1.08, 0.90–1.31, 0.397	1.04, 0.87–1.24, 0.649	1.09, 0.90–1.31, 0.381
	Never	1.22, 1.13–1.32, < 0.001	1.18, 1.07–1.29, < 0.001	1.23, 1.15–1.34, < 0.001	1.29, 1.18–1.42, < 0.001	1.18, 1.10–1.27, < 0.001	1.29, 1.18–1.40, < 0.001
TNM stage	I-II	1.59, 1.34–1.87, < 0.001	1.29, 1.17–1.42, < 0.001	1.67, 1.38–2.01, < 0.001	1.38, 1.12–1.69, 0.002	1.17, 0.84–1.62, 0.351	1.34, 1.09–1.65, 0.005
	III-IV	1.19, 1.13–1.27, < 0.001	1.18, 1.11–1.26, < 0.001	1.17, 1.10–1.23, < 0.001	1.26, 1.16–1.37, < 0.001	1.18, 1.09–1.27, < 0.001	1.24, 1.14–1.35, < 0.001
Lauren’s classification	Intestinal type	1.36, 1.18–1.57, < 0.001	1.27. 1.12–1.44, < 0.001	1.29, 1.11–1.51, 0.001	1.42, 1.20–1.66, < 0.001	1.21, 1.11–1.32, < 0.001	1.34, 1.14–1.58, < 0.001
	Diffuse type	1.19, 1.12–1.27, < 0.001	1.16, 1.07–1.26, < 0.001	1.17, 1.10–1.24, 0.001	1.13, 1.04–1.22, 0.003	1.12, 1.05–1.23, 0.003	1.16, 1.06–1.27, 0.001
Tumor embolus	Positive	1.23, 1.13–1.35, < 0.001	1.23, 1.14–1.33, < 0.001	1.28, 1.15–1.42, < 0.001	1.29, 1.15–1.44, < 0.001	1.16, 1.04–1.28, 0.006	1.27, 1.13–1.43, < 0.001
	Negative	1.17, 1.09–1.26, < 0.001	1.11, 1.03–1.21, 0.011	1.16, 1.08–1.23, < 0.001	1.25, 1.12–1.40, < 0.001	1.16, 1.06–1.28, 0.002	1.22, 1.09–1.37, 0.001
Obesity	With	1.11, 1.04–1.19, 0.031	1.13, 1.03–1.24, 0.012	1.09, 1.00–1.19, 0.051	1.12, 1.01–1.22, 0.042	1.07, 0.95–1.20, 0.276	1.07, 0.91–1.26, 0.399
	Without	1.26, 1.10–1.43, 0.001	1.18, 1.10–1.26, < 0.001	1.24, 1.09–1.40, 0.001	1.31, 1.20–1.43, < 0.001	1.21, 1.11–1.32, < 0.001	1.30, 1.18–1.42, < 0.001
Hypertension	With	1.07, 0.94–1.22, 0.338	1.05, 0.92–1.19, 0.502	1.08, 0.94–1.24, 0.271	1.17, 1.01–1.35, 0.037	1.14, 1.00–1.29, 0.045	1.15, 0.98–1.35, 0.096
	Without	1.22, 1.15–1.30, < 0.001	1.21, 1.14–1.28, < 0.001	1.21, 1.14–1.28, < 0.001	1.30, 1.18–1.42, < 0.001	1.16, 1.07–1.26, 0.001	1.27, 1.16–1.39, < 0.001
Diabetes	With	1.13, 1.04–1.22, 0.004	1.08, 1.02–1.16, 0.013	1.14, 1.05–1.25, 0.001	1.17, 1.08–1.28, 0.002	1.07, 0.98–1.16, 0.106	1.18, 1.08–1.29, < 0.001
	Without	1.18, 1.07–1.29, 0.001	1.16, 0.98–1.36, 0.080	1.15, 1.06–1.26, 0.001	1.21, 1.08–1.36, < 0.001	1.20, 1.05–1.38, 0.010	1.20, 1.09–1.30, < 0.001

Data are expressed as hazard ratio, 95% confidence interval, *P* value. Besides unadjusted overall estimates, the other risk estimates were calculated after adjusting for age, gender, smoking, drinking, family cancer history, body mass index, systolic blood pressure, diastolic blood pressure, fasting blood glucose, TNM stage, tumor size, Lauren’s classification, number of lymph node metastasis and tumor embolus by removing the characteristic itself in stratified analysis

*Abbreviations*: *AI* atherogenic index, *THR* the triglyceride to high-density lipoprotein cholesterol ratio, *LHR* the low-density lipoprotein cholesterol to high-density lipoprotein cholesterol ratio, *TNM* tumor-node-metastasis

By gender, the risk prediction of three lipid derivatives was markedly corroborated in male patients compared with female patients in both derivation and validation groups, especially for AI (HR, 95% CI, P: 1.27, 1.17–1.38, < 0.001 in the derivation group and 1.30, 1.18–1.43, < 0.001 in the validation group) and LHR (1.25, 1.15–1.36, < 0.001 in the derivation group and 1.26, 1.16–1.38, < 0.001 in the validation group). By smoking status, none or marginal significance was detected in ever smokers, while there was significant association between baseline three lipid derivatives and postoperative gastric cancer mortality, and risk estimates were comparable between both groups. By TNM stage, three lipid derivatives can better predict the significant risk of gastric cancer mortality in patients with stage I-II than those with stage III-IV, especially for AI (HR, 95% CI, P: 1.59, 1.34–1.87, < 0.001 versus 1.19, 1.13–1.27, < 0.001 in the derivation group) and LHR (1.67, 1.38–2.01, < 0.001 versus 1.17, 1.10–1.23, < 0.001 in the derivation group), and the prediction was reproducible in the validation group. By Lauren’s classification, there was significant prediction for three lipid derivatives, especially for intestinal-type gastric cancer, and the results were consistently reproduced in the validation group. By tumor embolus, the risk estimates were remarkably significant but slightly stronger in patients with positive embolus than in patients with negative embolus in both groups.

By obesity, the association of three baseline lipid derivatives, AI (HR, 95% CI, P: 1.26, 1.10–1.43, 0.001 versus 1.11, 1.04–1.19, 0.031 in the derivation group) and LHR (1.24, 1.09–1.40, 0.001 versus 1.09, 1.00–1.19, 0.051) with gastric cancer mortality was stronger in normal weight patients than in obese patients, and the same trend was observed in the validation group. By hypertension, risk was significant in patients without hypertension, and its magnitude was stronger than patients with hypertension in both groups. By diabetes, only AI and LHR exhibited a significant association with gastric cancer mortality, which was slightly reinforced in patients without diabetes, and the derived results were reproduced in the validation group (Table 2).

Although the majority of comparisons were reproducible between the derivation group and the validation group, there were some exceptions, for example, the risk prediction of THR and LHR for gastric cancer prognosis in female patients was significant in the derivation (HR, 95% CI, P: 1.17, 1.03–1.33, 0.019 and 1.19, 1.10–1.29, < 0.001) but not in the validation group (1.12, 0.97–1.29, 0.136 and 1.17, 0.96–1.42, 0.118), possibly due to insufficient statistical power as there were respectively 410 (27.22%) and 363 (24.1%) female patients involved in the derivation and validation groups.

### Kaplan-Meier survival analysis

**Fig. 1:**
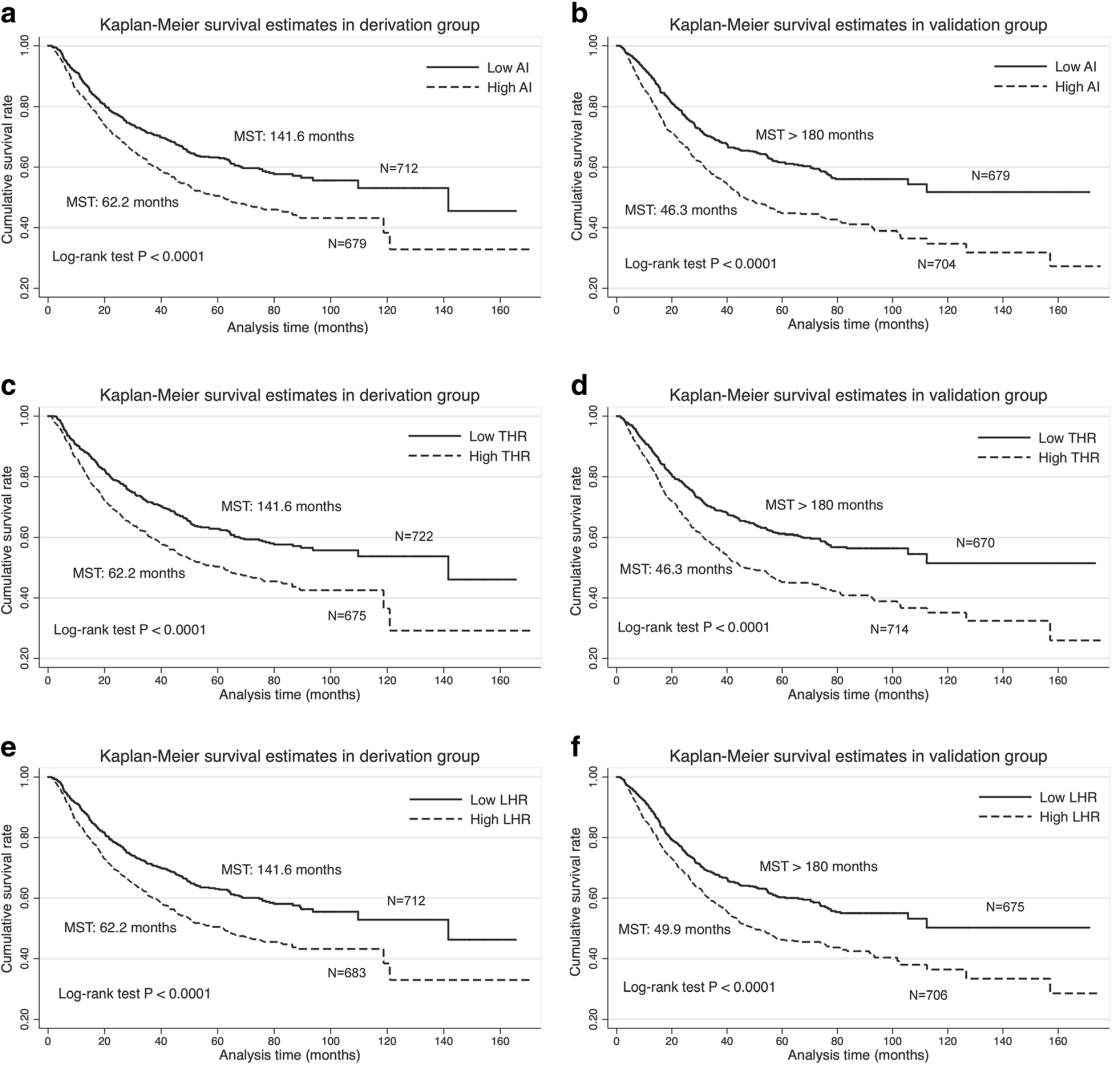
Kaplan-Meier curves for binary AI (**a** and **b**), THR (**c** and **d**) and LHR (**e** and **f**) in both derivation and validation groups. Abbreviations: AI, atherogenic index; THR, triglyceride to high-density lipoprotein cholesterol ratio; LHR, low-density lipoprotein cholesterol to high-density lipoprotein cholesterol ratio; MST, median survival time

The median values of AI, THR and LHR were 3.41, 0.97 and 2.89 among all study patients, respectively, and they were used as binary cut-off thresholds in Kaplan-Meier survival curves (Fig. 1). Patients with lower levels of AI (Fig. 1a and b), THR (Fig. 1c and d) and LHR (Fig. 1e and f) separately had better survival rates than those with the corresponding higher levels in both derivation and validation groups. In addition, MST was significantly longer in patients with lower levels of AI, THR and LHR than those with the corresponding higher levels (Log-rank test *P* < 0.0001 for all comparisons) in both groups.

### Calibration and discrimination

Calibration and discrimination abilities for the addition of individual lipid derivatives to the baseline risk model in predicting gastric cancer mortality are presented in Table 3. Both AIC and BIC statistics were reduced by more than 10 after the addition of AI and LHR to the baseline risk model in both derivation and validation groups. No material changes in both statistics were noted for THR. In addition, the − 2 log likelihood ratio test was markedly significant for AI and LHR in both groups, indicating that AI and LHR were indeed a part of true model and carried a better fit, while there was only marginal significance for THR.

**Table 3:** Predictive accuracy of baseline risk model and the addition of three individual lipid derivatives for gastric cancer mortality in both derivation and validation groups

Statistics	Derivation group (*n* = 1506)				Validation group (*n* = 1506)			
	BR-Model (BRM)	BRM plus AI	BRM plus THR	BRM plus LHR	BR-Model (BM)	BRM plus AI	BRM plus THR	BRM plus LHR
AIC	2476	2461	2473	2458	2604	2584	2599	2586
BIC	2532	2523	2531	2519	2660	2645	2660	2647
LR test: [Chi]^2^	Reference	16.26	4.9	17.85	Reference	12.58	5.34	12.55
LR test: P	Reference	0.0001	0.0269	< 0.0001	Reference	0.0004	0.0208	0.0004
Harrell’s C	0.7662	0.7693	0.7679	0.7697	0.7541	0.7587	0.7555	0.7579

BR-Model included age, gender, smoking, drinking, family cancer history, body mass index, systolic blood pressure, diastolic blood pressure, fasting blood glucose, tumor-node-metastasis stage, tumor size, Lauren’s classification, number of lymph node metastasis and tumor embolus

*Abbreviations*: *BR-Model (BRM)* baseline risk model (BRM), *AI* atherogenic index, *THR* the triglyceride to high-density lipoprotein cholesterol ratio, *LHR* the low-density lipoprotein cholesterol to high-density lipoprotein cholesterol ratio, *AIC* Akaike information criterion, *BIC* Bayesian information criteria, *LR test* likelihood ratio test

As indicted by the Harrell’s C-statistic, baseline risk model and modified models by separately adding three individual lipid derivatives had better predictive accuracy in both derivation and validation groups.

### Decision curve analysis

**Fig. 2:**
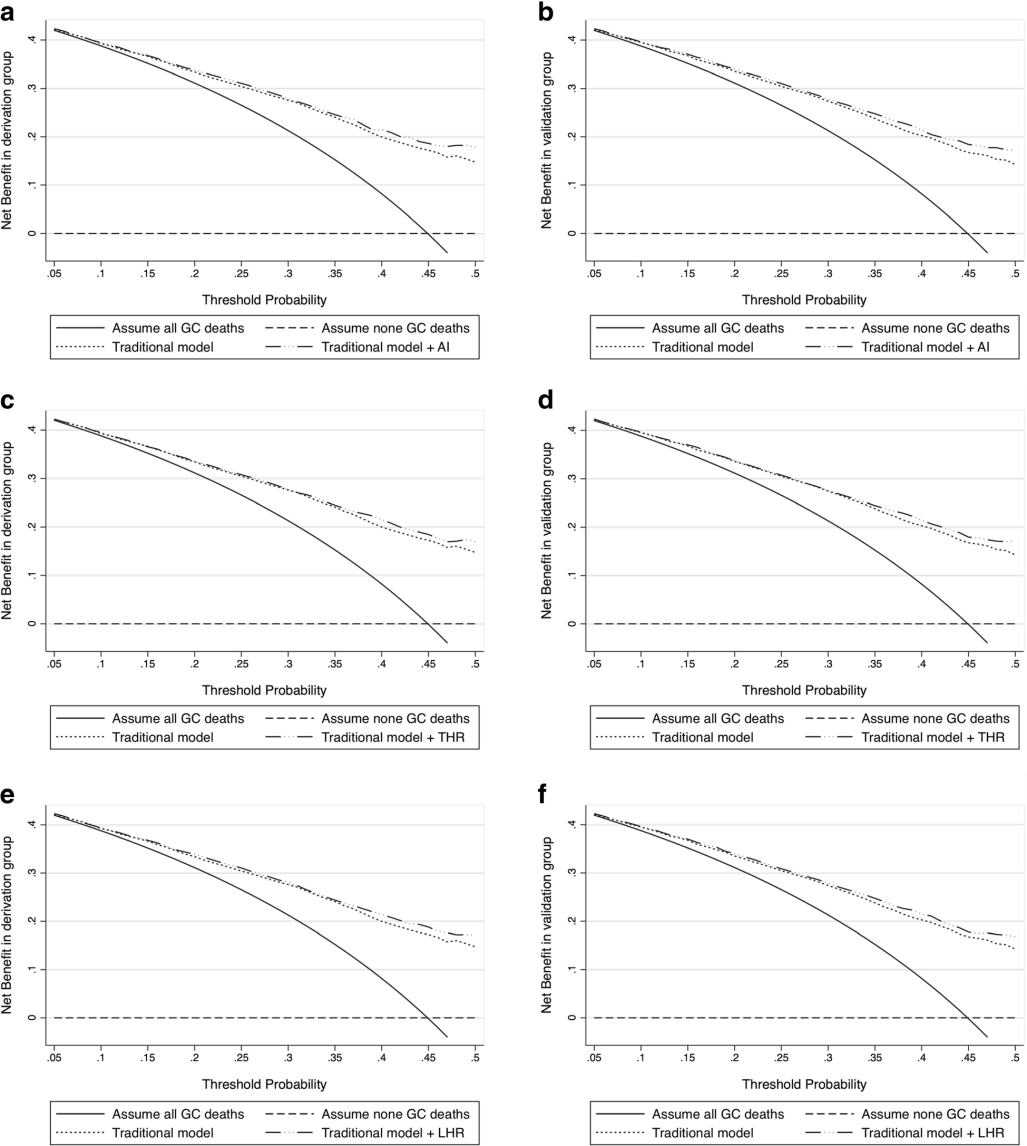
Decision curves for baseline risk model (termed traditional model) and the addition of AI (**a** and **b**), THR (**c** and **d**) and LHR (**e** and **f**) in both derivation and validation groups. Abbreviations: AI, atherogenic index; THR, triglyceride to high-density lipoprotein cholesterol ratio; LHR, low-density lipoprotein cholesterol to high-density lipoprotein cholesterol ratio; GC, gastric cancer. Baseline risk model included age, gender, smoking, drinking, family cancer history, body mass index, systolic blood pressure, diastolic blood pressure, fasting blood glucose, tumor-node-metastasis stage, tumor size, Lauren’s classification, number of lymph node metastasis and tumor embolus

Based on decision curve analysis, gained usefulness of individual lipid derivatives over the baseline risk model is presented in Fig. 2. Gained net benefits by adding AI (Fig. 2a and b), THR (Fig. 2c and d) and LHR (Fig. 2e and f) were higher than that of the baseline risk model in both derivation and validation groups.

### Prognostic nomogram

**Fig. 3:**
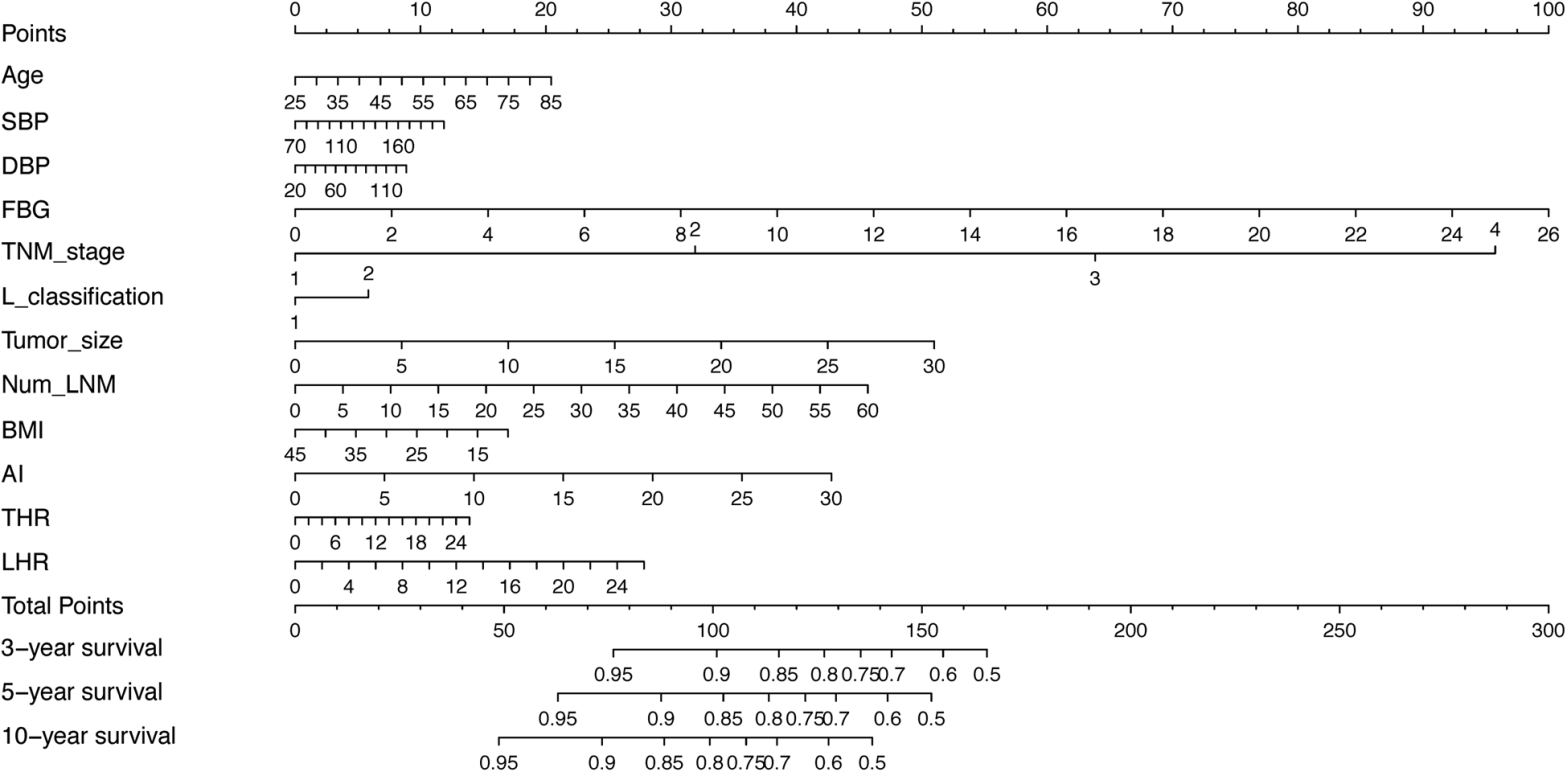
Prognostic nomogram for the prediction of significant characteristics along with AI, THR and LHR for 3-year, 5-year and 10-year survival of all gastric cancer patients. Abbreviations: SBP, systolic blood pressure; DBP, diastolic blood pressure; FBG, fasting blood glucose; TNM, tumor-node-metastasis; L_classification, Lauren’s classification; BMI, body mass index; AI, atherogenic index; THR, triglyceride to high-density lipoprotein cholesterol ratio; LHR, low-density lipoprotein cholesterol to high-density lipoprotein cholesterol ratio. Predictive magnitude can be calculated by drawing a vertical line linking the value of each parameter with the point score at the top of this nomogram (the “Points” line). Next, the individual scores are summed to generate a total point score, which is plotted along the “Total Points” line, as well as the “3-year survival”, “5-year survival” and “10-year survival” line at the bottom of this nomogram to judge the risk of gastric cancer mortality

A nomogram was depicted to predict 3-year, 5-year and 10-year individualized absolute risk for gastric cancer mortality based on significant attributes among all study patients (Fig. 3). Significant attributes were selected from baseline demographic, clinical and clinicopathologic characteristics by the Weibull proportional hazards model, including age, systolic BP, diastolic BP, FBG, TNM stage, Lauren’s classification, tumor size, number of LNM, BMI, AI, THR and LHR (all *P* < 0.05). Predictive accuracy of this nomogram was good, with the C-index of being 0.773. Calibration curves for 3-year, 5-year and 10-year survival prediction indicated good agreement between predicted probabilities by this nomogram and actual observations (Additional file 1: Figure S1).

## Discussion

Extending our previous findings on metabolic syndrome and gastric cancer [9], we evaluated the prediction of three lipid derivatives for the prognosis of postoperative gastric cancer. Importantly, our findings indicate that lipid derivatives, especially AI and LHR, are powerful predictors of gastric cancer mortality, and the prediction is more obvious in patients of male gender or with TNM stage I-II or intestinal-type gastric cancer, and in the absence of obesity or hypertension before gastrectomy. A revisit to derived components of the metabolic syndrome would be an essential step toward better understanding of metabolomics in the pathophysiology of gastric cancer, and facilitate the development of new therapeutic targets.

Recently, Xiao and Zhou have written an excellent review about the metabolism and metabolomics of gastric cancer, and they underscored the close relevance between metabolic changes and gastric carcinogenesis, implicating the metabolism-based antitumor therapies in future clinical practice [25]. In this context, some studies have investigated the influence of the metabolic syndrome and its components on the risk of developing gastric cancer [26–29]. However, less research on the prediction of coexisting metabolism syndrome for postoperative survival of gastric cancer has been done so far. For example, Wei and colleagues reported that gastric cancer patients complicated with the metabolism syndrome had improved tumor cell differentiation and had a better chance of postoperative survival [30]. Another study by Kim and colleagues revealed that coexistence of the metabolic syndrome can increase the risk of gastric cancer mortality after receiving gastrectomy [31], as consolidated by our recent FIESTA study in a prospective analysis of 3012 gastric cancer patients over a 15-year follow-up period [9]. Hence, exhaustive research is still needed to address these inconsistencies to enable precise clinical translation.

It is well known that the metabolic syndrome is the collective term given to a group of risk factors that evoke cardiovascular disease and diabetes, and abnormalities in these factors will aid clinical management of the metabolic syndrome as an efficient therapeutic strategy to improve survival outcomes of gastric cancer patients [32, 33]. In particular, dyslipidemia has been widely recognized as a diagnostic tool for the metabolic syndrome [34]. According to the criteria proposed by the Chinese Diabetes Society (2004) [35], dyslipidaemia is contingent on a simple assembly of TG and HDLC, and it disregards the other two blood lipids – TC and LDLC. To provide an in-depth evaluation of dyslipidemia, we developed three ratio derivatives based on the four blood lipids, and plenty of studies have pointed out these three lipid derivatives as more effective predictors of cardiovascular diseases than individual lipids [10, 11]. However, it is rarely reported about the predictive role of these lipid derivatives in the development and progression of gastric cancer. The present study attempted to fill the gap on this topic by revisiting the FIESTA database [9, 16] to evaluate the long-term prediction of three lipid derivatives at baseline for the prognosis of 3012 gastric cancer patients after gastrectomy. We importantly found that three lipid derivatives, especially AI and LHR, were powerful predictors of gastric cancer mortality, and the predictive performance was augmented in male or early stage patients or with coexistent obesity or hypertension at baseline. From a clinical perspective, our findings were biologically meaningful. It has been proposed that lipid abnormalities, especially reduced HDLC, play an important role in carcinogenesis through antioxidant and anti-inflammatory properties [36, 37]. HDLC was reported to modulate angiogenesis, a critical biological process that is altered during carcinogenesis, in a multifunctional manner, depending on pathophysiological context [38]. In addition, other potential biologic mechanisms might involve the changes of endogenous hormones such as sex steroids associated with obesity and the contribution of obesity to gastric carcinogenesis [39, 40]. Exploring the underlying molecular mechanisms of lipid abnormalities in gastric carcinogenesis is beyond the scope of our present study but certainly requires further investigation. So, we propose that clinical management of lipid abnormalities, especially HDLC, can help prolong survival and improve quality of life for patients with resectable gastric cancer.

From a statistical perspective, our findings are reproducible (with few exceptions), reliable and applicable. In fact, we adopted a two-phase sampling design by evenly randomizing study patients into the derivation group and the validation group. Of note, all effect-size estimates generated in the derivation group were consistently reproduced in the validation group, underscoring the robustness of these lipid derivatives. In addition, our findings have been proven to bear high reliability, as multiple calibration and discrimination statistics consistently indicated that adding individual lipid derivatives to baseline risk model had better classification performance and predictive accuracy. Furthermore, in view of strong predictive power of three lipid derivatives, we established a prognostic nomogram as a calculator to predict 3-year, 5-year and 10-year individualized absolute risk, and this nomogram had good predictive accuracy, as reflected by both C-index and calibration curves. Hence, our findings can be easily applied for risk assessment in routine clinical practice.

Finally, several limitations should be acknowledged in this study. First, this study was carried out in a mono-centre, and the findings could be generalizable pending consistently validated in other studies and cohorts. Second, the effects of lipid-lowering agents on the prognosis of gastric cancer patients remained unexplored, as such data were currently lacking, which might generate a systematic bias and unaccounted residual confounding. Third, all study patients were recruited between January 2000 and December 2010, and during such a long period, remarkable advances in surgical techniques might introduce a possible bias. Fourth, our findings were based on only gastric cancer patients who received gastrectomy, and thereby cannot be extrapolated to the general patient populations. Nonetheless, not only does lipid-lowering treatment have significant clinical implications in gastric cancer patients, but it is necessary for future continued investigations to unravel the molecular mechanisms linking dyslipidaemia with poor prognosis for precise therapeutic intervention and finding additional drug targets, which will have considerable public health significance.

## Conclusions

Based on an in-depth analysis, our findings indicate that preoperative lipid derivatives, especially AI and LHR, are powerful predictors of gastric cancer mortality, and the prediction is more obvious in patients of male gender or with TNM stage I-II or intestinal-type gastric cancer, and in the absence of obesity or hypertension before gastrectomy. For practical reasons, an increased understanding of lipid abnormalities in gastric carcinogenesis can provide the foundation to facilitate the development of therapeutic agents that may identify novel applications for future clinical testing in postoperative gastric cancer patients.

## Additional file

Additional file 1: **Figure S1.** Calibration curves for predicting the risk of gastric cancer mortality at 3 years (A), 5 years (B) and 10 years (C) among all gastric cancer patients. Nomogram-predicted probability of overall survival is plotted on the X-axis, and actual overall survival is plotted on the Y-axis. (PDF 79 kb)

## Abbreviations

95% CI: 95% confidence interval
AI: Atherogenic index
AIC: Akaike information criterion
BIC: Bayesian information criterion
BMI: Body mass index
BP: Blood pressure
C-index: Concordance index
FBG: Fasting blood glucose
FIESTA: Fujian Prospective Investigation of Cancer
HDLC: High-density lipoprotein cholesterol
HR: Hazard ratio
LDLC: Low-density lipoprotein cholesterol
LHR: The ratio of LDLC to HDLC
LNM: Lymph node metastasis
MST: Median survival time
RMS: Regression modeling strategies
SD: Standard deviation
TC: Total cholesterol
TG: Triglycerides
THR: The ratio of TG to HDLC
TNM: Tumor-node-metastasis
