# Supplementary figures and images for: Prediction of three lipid derivatives for postoperative gastric cancer mortality: the Fujian prospective investigation of cancer (FIESTA) study
Source: BMC Cancer. 2018 Aug 6;18:785. doi: 10.1186/s12885-018-4596-y (PMC6080391; doi:10.1186/s12885-018-4596-y)

Figure S1

(A)

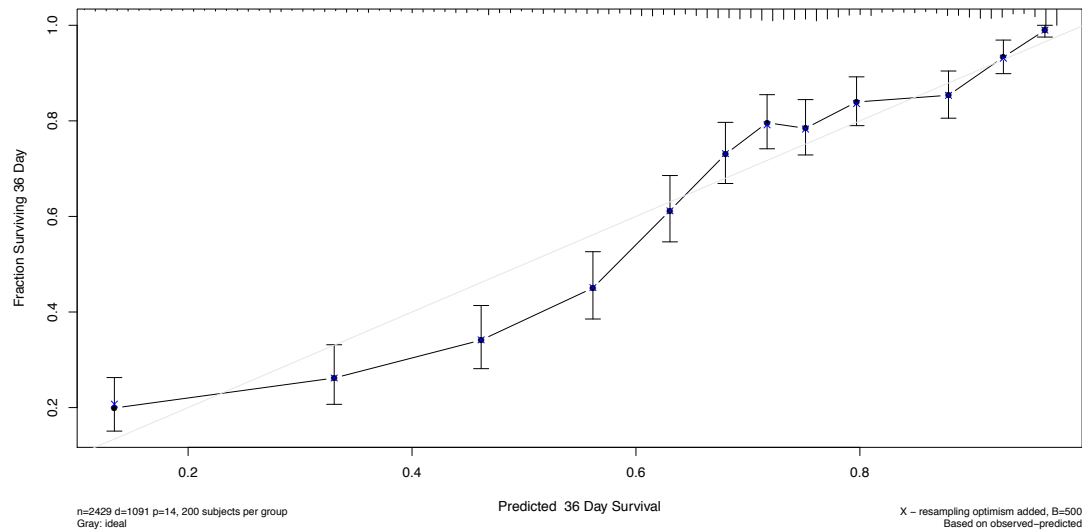

(B)

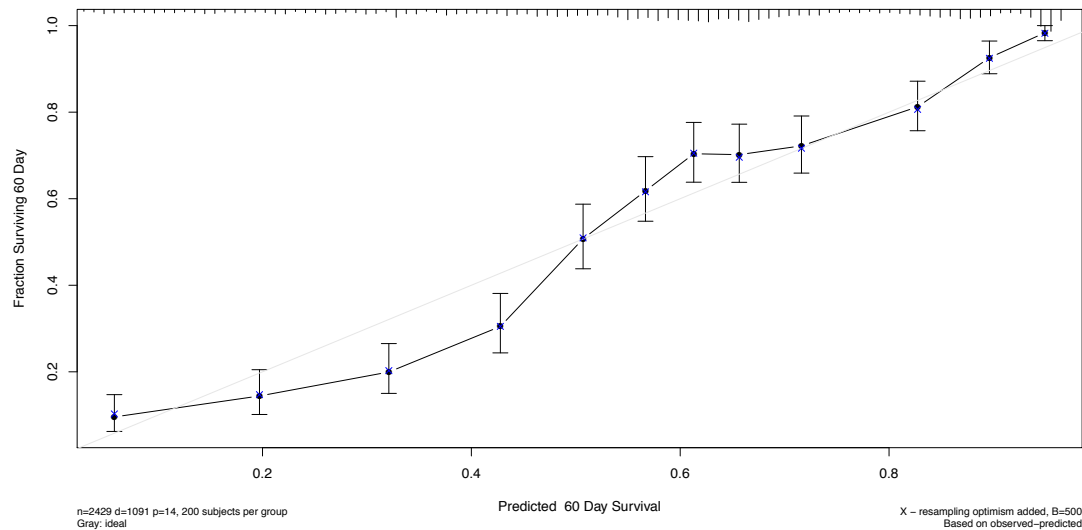

(C)

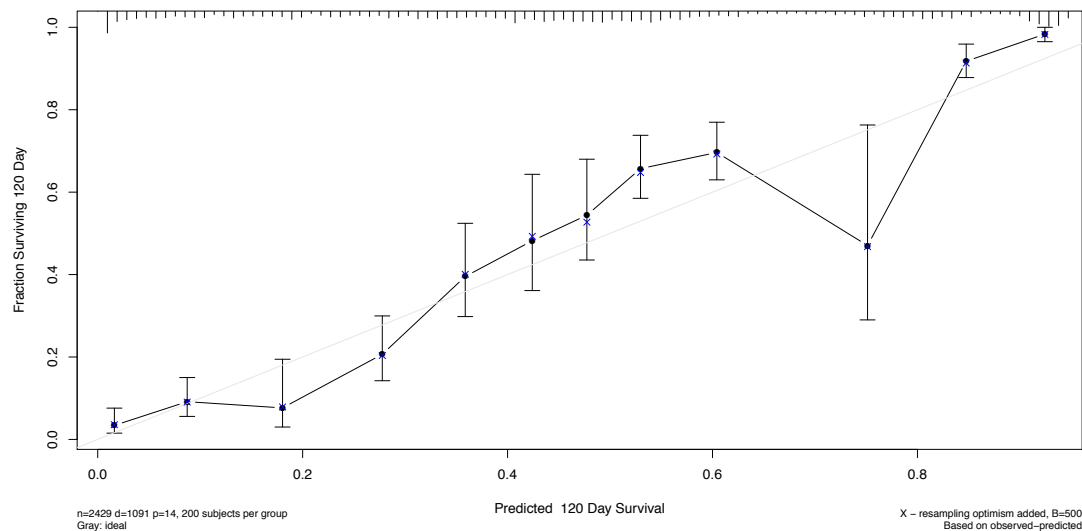

Supplement: Supplementary file 1 — Figure S1. Calibration curves for predicting the risk of gastric cancer mortality at 3 years (A), 5 years (B) and 10 years (C) among all gastric cancer patients. Nomogram-predicted probability of overall survival is plotted on the X-axis, and actual overall survival is plotted on the Y-axis. (PDF 79 kb) [file 12885_2018_4596_MOESM1_ESM.pdf]
